# The effect of *Amygdalus scoparia Spach* and *Lepidium sativum* L. seed gums on the properties of formulated food supplement for soldiers using Response Surface Methodology

**DOI:** 10.1002/fsn3.2207

**Published:** 2021-02-26

**Authors:** Amirhossein Razjoo, Maryam Azizkhani, Reza Esmaeilzadeh Kenari

**Affiliations:** ^1^ Department of Food Hygiene Faculty of Veterinary Medicine Amol University of Special Modern Technologies Amol Iran; ^2^ Department of Food Science and Technology Sari Agricultural sciences and Natural Resources University (SANRU) Sari Iran

**Keywords:** energy intake, food supplement, shahi gum, zedo gum

## Abstract

Today, the lack of a proper nutritional formulation of the diet for soldiers is well felt. In this study, a newly formulated food supplement (FFS) was designed to supply all essential nutrients like protein, carbohydrate, oil, fat‐soluble vitamins, and minerals, and *Lepidium sativum* L. seed (shahi) gum and A*mygdalus scoparia Spach* (zedo) gum were applied to FFS to promote physicochemical and sensory properties of FFS. The samples were developed by preparing emulsion including meat powder (45.0 g/100 g), soybean powder (25.0 g/100 g), and plant oils (15.0 g/100 g). Iron, zinc, and fat‐soluble vitamins (A, D, E, and K) were also added to the formulation, and response surface methodology was used to optimize the effects of shahi and zedo gum at 0.5, 1.0, and 2.0 g/100 g in FFS. The results showed that using hydrocolloids in appropriate amounts enhanced the sensory properties of FFS. Hydrocolloids protected the moisture content of FFS samples and also decreased the reduction of vitamins and minerals during 14 days of storage at 4°C. The hydrocolloids improved the color indices and intrinsic viscosity of samples. The results of this study recommend the use of 1.26 g/100 g of shahi gum and 0.95 g/100 g of zedo gum to produce formulated food supplement for soldiers. This formulation supplies calories and provides some of the essential vitamins and food components to the body.

## Graphical abstract

In this study, a new formulated food as a supplement for soldiers was designed according to the emulsification method, the properties of food formulation were optimized by adding native gums using response surface methodology, and the change in the properties of formulated food during 14 days of storage was investigated. The findings of this study would be useful to improve the food health and quality of the military diet and extend the new field of high‐energy fortified foods.

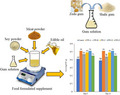

**Highlights**

- Using hydrocolloids enhanced the sensory properties of fortified food.
- Hydrocolloids decreased the reduction of vitamins and minerals during storage.
- Hydrocolloids improved the color indexes and intrinsic viscosity of samples.

## 1 INTRODUCTION

The relation between physical performance and good nutrition is well recognized in organizations like the Army, where mentally and physically demanding jobs are standard (Jayne et al., 2018). Besides the physical and mental supports, nutrition and hydration are important factors that provide conduction of military actions (Ciapa, 2020). Promoting adherence to army standards occurs as a result of the healthy food choice, positively affect multiple body systems influencing overall health (Jayne et al., 2018). The lack of energy supply caused by the insufficient amount of food and water, reduced time for food consumption, and reduced appetite due to stress factors (Ciapa, 2020) can be compensated by consumption of formulated food supplement (FFS) containing all nutrient intakes like protein, polyunsaturated fatty acids, carbohydrate, fat‐soluble vitamins, and minerals.

Proteins accelerate the chemical reaction in the body, maintain and strengthen its structure, distribute oxygen throughout it, fight against its infection, and serve as its chemical messengers. Among proteins, animal proteins are considered complete proteins because they provide all of the essential amino acids our bodies need (Rostami et al., 2018). Soybean protein includes all essential amino acids that are important for health, and they were suggested as highly digestible (Taghdir et al., 2017). Soybean powder also contains a high concentration of antioxidants like isoflavones, genistein, and daidzein and rich in micronutrients (Jalgaonkar et al., 2018; Tufa et al., 2016). Soybean powder has been used to improve the quality, shelf life, and nutritional value of foods (Taghdir et al., 2017; Tufa et al., 2016). Rice bran oil contains unique bioactive compounds, and it has high polyunsaturated fatty acid and gamma‐oryzanol (Chompoo et al., 2019). Sesame seed oil is unique with a remarkable amount of sesamol, sesamolin, and sesamin which exhibited antioxidant properties (Chompoo et al., 2019). Canola oil is characterized by substantial amounts of polyunsaturated fatty acids, plant sterols, and tocopherols and is recommended for the treatment of cardiovascular diseases (Lin et al., 2013).

Gums are a wide range of hydrophilic biopolymers, employed in food processing to produce stable emulsions, thicken solution, and functional foods (Seyfi et al., 2019). Polysaccharides improve rheological properties, retain the moisture, preserve flavor, extend shelf life, stabilize and emulsify food systems, and add value to food products (Seyfi et al., 2019). Zedo (*Amygdalus scoparia Spach*) is cultivated in different regions of Iran (Gharanjig et al., 2020; Seyfi et al., 2019). The exudate gum contains l‐arabinose, d‐xylose, d‐galactose, and uronic acid monosaccharides (Gharanjig et al., 2020). Shahi (*Lepidium sativum* L.) belongs to the Brassicaceae family, which is mostly cultivated in Iran. Shahi seed gum contains l‐arabinose, d‐xylose, d‐galactose, l‐rhamnose, d‐glucuronic acid, and d‐glucuronic acid (Gharanjig et al., 2020).

Formulated products are special categories of foods (Taifouris et al., 2020), which can be obtained using engineering and mathematical formulation. Production of novel emulsion particles, food structure, sustainable food systems, modern food supply, considering human health and well‐being, and innovation in food formulation is possible using food engineering (Roos et al., 2016). Until now, no study was conducted to design comprehensive food supplements for soldiers which provide all essential nutrients and calories. Also, despite the increase in nutritional needs of soldiers in terms of amount, type and timing, all essential dietary intakes are not available in a critical situation like war or military bases away from the city, and extra supplementation via food extras is required. Therefore, the purpose of this study was to (a) design new formulated food as a supplement for soldiers according to the emulsification method, (b) optimize the properties of food formulation by adding native gums using response surface methodology, and (c) investigate the change in properties of formulated food during 14 days of storage.

## 2 MATERIALS AND METHODS

### 2.1 Materials

Chicken meat powder (protein = 82.4%, fat = 11.2%) and soy powder (fat = 21.0%, protein = 34.2%, and carbohydrate = 36.1%) were purchased from Kimia‐Tejarat (Alborz, Iran). Zedo and shahi gum were purchased from Reyhan gum Parsian (Sari, Iran). Sesame seed oil, rice bran oil (Zarin‐talia Co.), and canola oil (Oila Co.) were purchased from the local market. All other chemicals were of analytical grade and bought from Sigma‐Aldrich.

### 2.2 Methods

#### 2.2.1 Production of formulated food supplement (FFS)

FFS samples were prepared by the emulsification techniques. For this purpose, fat‐soluble vitamins, salts, and minerals were added to the oil mix. Then, tween 80 emulsifier (0.5 g) was added to the mixture under stirring conditions at room temperature (25°C for 30 min). The powder of meat and soybean gradually were added to the oil base mixture, and they were thoroughly mixed using an electrical mixer (JJ‐5, Hongda, China) for 10 min. To prepare gum solutions (Table 1) separately 0.5, 1.0, or 2.0 g of different gums were added to 14, 13.5, and 12.5 g of water, respectively, under continuously stirring at room temperature. The control was prepared by the same method applied for the samples but without gums, and water was used instead of gums. The final weight of different FFS formulation samples, regardless the weight of the minerals and vitamins, was 100 g. After complete dissolution of gums, the solutions were added to the food mixture and stirred for 2 hr. The FFS samples were homogenized at 49,200 g for 1 min by using high speed homogenizer (ULTRA‐TURRAX T18 IKA) to ensure emulsion homogeneity. The prepared FFS samples were stored at 4°C, and different tests were done at days 0 and 14 of the storage time on optimized samples. Table 1 shows the amount of ingredients in FFS samples.

**TABLE 1:** The amount of ingredients in FFS

Ingredient	Amount (100 g)
Meat powder	45.0 g
Soy powder	25.0 g
Canola oil	12.0 g
Sesame seed oil	1.5 g
Rice bran oil	1.5 g
Sodium chloride	0.5 g
Vitamin E	1.0 g
Vitamin A	3.0 mg
Vitamin K	50.0 µg
Vitamin D	50.0 µg
Iron	20.0 mg
Zinc	30.0 mg
Zedo or shahi gum	0.5, 1, or 2 g
Water	14, 13.5 or 12.5 g

#### 2.2.2 Calorie content

To calculate the calorific value of FFS samples, approximately 1 g of dried (oven drying method) sample was tested in the calorimetry bomb (C‐200, Ika, Germany). The calorific values were expressed in calories per 10 g (Razavi et al., 2020).

#### 2.2.3 Intrinsic viscosity

Intrinsic viscosity was determined from 1 to 100 cm^3^/g at 25°C using an automatic viscometer (Lauda‐Königshofen, Germany) equipped with an Ubbelohde capillary tube and appropriate spindle. An average viscosity at 50 cm^3^/g was reported as the intrinsic viscosity.

#### 2.2.4 Color analysis

The color of control and FFS samples was evaluated by determining three parameters of L*, a*, and b* using Minolta Hunterlab (C360, Japan) through the CIE method. L* represents the lightness, a* represents the redness/greenness quality of the color, and b* represents the yellowness/blueness quality of the color (Chaves et al., 2018).

#### 2.2.5 Moisture content

The moisture content was determined for each FFS sample as the percentage ratio of the weight loss to the initial weight of the sample as in Equation 1. below (AOAC, 2006). Samples were dried at 105°C for 5 hr.

(1) $$\mathrm{MC}=\frac{\left(W_{i}‐W_{f}\right)}{\left(W_{f}\right)}\times 100$$

*W_i_* = initial weight; *W_f_* = final weight, and MC = the moisture content.

#### 2.2.6 Mineral and vitamins determination

Analysis of iron and zinc content of the FFS samples was carried out using atomic absorption spectrophotometer (Z6100, Hitachi, Tokyo, Japan) equipped with a PC‐controlled 6‐piece lamp turret. Hollow cathode lamps (248.3 nm for Fe and 213.85 nm for Zn) were mounted as line radiator along with a deuterium lamp for neutralization of the background absorption. Signal measurements were done in peal area/peak height. Briefly, 1 g of the sample was digested in 20 ml of HNO_3_ and HCl at 1:1 v/v ratio and after cooling to room temperature diluted to 100 ml using distilled water. The stock solutions of Fe and Zn were prepared to obtain standard curves. (AOAC, 2006). Fat‐soluble vitamins were measured using reversed‐phase high‐performance liquid chromatography (HPLC) with diode detection (Sharma et al., 2020).

#### 2.2.7 Sensory evaluation

A 5‐point hedonic scale (Scale: 1‐dislike extremely; 2‐dislike slightly; 3‐neither like nor dislike; 4‐like slightly; 5‐like extremely) was applied for sensory evaluation of the samples. A group of 10 trained panelists (five women, five men), ages between 30–60, were chosen to evaluate sensory properties of the samples focusing on the color, taste, odor, and appearance. Samples were provided in 30‐ml cups, and prior to the organoleptic evaluation, water was used for mouth rinsing.

#### 2.2.8 Experimental design

A 2‐factor and 3‐level central composite design was applied for investigating the effect of two independent variables, concentration, and type of gums, on the calorie value and viscosity of the formulated food samples as responses. The ranges studied were 0.0–2.0 g/100 g for zedo gum (ZD) and 0.0–2.0 g/100 g for shahi gum (SH). Ten experimental settings (eight factorial points and two center points) were generated using the Design of Expert software. The experimental design is presented in Table 2. Experiments were randomly run, and triplicate analyses were performed at each design point.

**TABLE 2:** Central composite design and responses

Experiment no	Uncoded (coded) levels		Calorie (Cal/100 g)		Viscosity at 50/s (Pa s)	
	Shahi	Zedo	Observed	Predicted	Observed	Predicted
1	0 (−1)	1 (0)	249.69	249.74	0.152	0.150
2	2 (+1)	2 (+1)	257.44	255.06	1.73	1.73
3	1 (0)	0 (−1)	250.13	252.15	0.707	0.705
4	1 (0)	1 (0)	253.00	252.69	0.835	0.838
5	0 (−1)	2 (+1)	255.10	256.43	2.11	2.12
6	1 (0)	2 (+1)	255.31	248.12	1.89	1.91
7	2 (+1)	0 (−1)	252.68	255.76	1.08	1.06
8	+1 (0)	1 (0)	248.12	253.04	0.998	0.996
9	2 (+1)	1 (0)	256.79	255.32	1.35	1.33
10	0 (−1)	0 (−1)	247.58	247.58	0.512	0.511

Ten experimental settings (eight factorial points and two center points) were generated using the Design of Expert software.

### 2.3 Statistical analysis

In this study, treatment means were compared using one‐way analysis of variance (one‐way ANOVA) and Duncan's multiple range test at 5% level of significant analysis by SPSS (Version 20.0, Chicago, SPSS Inc). All the experiments were carried out in triplicates.

## 3 RESULTS AND DISCUSSION

### 3.1 Optimization of treatments using viscosity and calorie values

The central composite design, the levels of factors, and the amount of calorie in the FFS samples prepared using different levels of gums are shown in Table 2. The calorie value of the samples ranged from 242.06 to 277.03 Cal. The response model equation for the calorie can be written as follows (Equation 2):

(2) Y=+0.247.19+2.754shahi+2.910zedo

**FIGURE 1:**
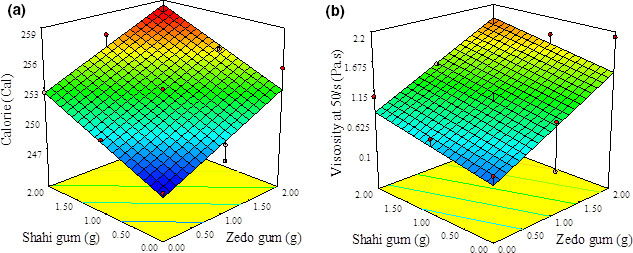
3d response surface plots of interaction between shahi gum with zedo gum on a) calorie (Cal) and b) viscosity (Pa s)

As shown in Table 3, shahi gum was the most significant independent variable. In other words, in equal *p*‐value, the variables that have higher *F*‐value were more significant than others. The linear term of shahi and zedo gum positively impacted the calorie. As shown in Figure 1a, the calorie of the FFS samples increased as gum content increased. The results of calorific value of the optimized FFS samples are shown in Table 4. However, no statistically significant difference was observed among the samples (*p* < .05), but the control samples showed the least calorific value. According to this matter that energy requirements of military personnel are about 2.3–7.1 kcal based on energy consumption and need (Ciapa, 2020), eating 4 cups of FFS in addition to providing minimum required amount of the energy eliminates the need for essential nutrients such as vitamins, iron, zinc, proteins, and essential fatty acids. According to the results, five optimized formulations which had higher calorie were chosen and prepared by varying shahi and zedo gum content based on Table 4.

**TABLE 3:** Analysis of variance (ANOVA) in linear model

Factor	*df*	Sum of square	Mean square	*F*‐value	*p*‐value
Calorie (Cal)					
Model	2	103.15	51.57	31.59	.00*
Shahi gum	1	52.24	52.24	32.05	.00*
Zedo gum	1	50.81	50.81	31.12	.00*
Residual	7	11.43	1.63		
Lack of fit	6	10.20	1.70	1.38	.57
Pure error	1	1.23	1.23		
Viscosity at 50/s (Pa s)					
Model	2	2.30	1.15	6.33	.02*
Shahi gun	1	0.34	0.34	1.85	.21
Zedo gum	1	1.96	1.96	10.80	.01*
Residual	7	1.27	0.18		
Lack of fit	6	0.91	0.15	0.43	.82
Pure error	1	0.36	0.36		

* Values are significant at 95% confidence level.

**TABLE 4:** Calorific value and viscosity of optimized samples

Code	Shahi gum (g/100 g)	Zedo gum (g/100 g)	Calorific value (Cal/100 g)	Viscosity at 50/s (Pa s)
Control	0.00	0.00	242.06 ± 5.05^a,^ *	0.51 ± 0.01^d^
FFS‐A	1.26	0.95	266.06 ± 6.80^a^	1.74 ± 0.05^b^
FFS‐B	0.79	1.04	243.0 ± 5.68^a^	1.57 ± 0.05^c^
FFS‐C	0.99	1.17	252.86 ± 6.27^a^	1.82 ± 0.04^a^
FFS‐D	0.92	1.23	277.03 ± 8.75^a^	1.84 ± 0.02^a^
FFS‐E	0.44	1.21	262.58 ± 7.13^a^	1.54 ± 0.05^c^

* Different lowercase letter indicates the significant statistical differences at *p* < .05.

The intrinsic viscosities of the FFS samples at 50 s^−1^ are shown in Table 2. The viscosity of samples ranged from 0.152 to 2.12 Pa s. The *p*‐values of the linear model are shown in Table 3. The response model equation for the viscosity can be written as follows (Equation 3):

(3) Y=+0.367+0.220shahi or zedo+0.571

The analysis of variance (Table 3) revealed that the linear model showed no lack of fit value (*p* > .05), and zedo gum was more significant than shahi gum. As shown in Figure 1b, the viscosity increased with increasing gum concentration. One of the most considerable features of polysaccharides is the increase in viscosity despite being used in very small quantity (Fadavi et al., 2017). Viscosity is a parameter to analyze or characterize colloid or emulsion systems. The results of the viscosity of optimized FFS samples are presented in Table 4.

Control samples, without any gums, showed the least viscosity. As shown in Table 4, the viscosity of FFS samples increased with the increase of their gum concentration and the type of gums affected the viscosity. Zedo gum has a more significant effect than shahi on increasing the viscosity of FFS samples. Fadavi et al. (2017) reported that an increase in zedo solution from 1.0% to 5.0% w/v caused an increase in intrinsic viscosity (Fadavi et al., 2017). It seems that higher amounts of zedo gum interfere with the formation of a protein network, decrease the phase separation in FFS, and lead to viscosity increment. These results are in agreement with the results of Alizadeh Khaledabad et al. (2020), who reported the use of zedo gum and enhanced the apparent viscosity of yoghurt (Alizadeh Khaledabad et al., 2020). Farahmandfar et al. (2017) showed that the addition of shahi seed gum hydrocolloid and an increase in its concentration caused a decrease in the fluidity of whipped cream samples. It is attributed to the binding of water by hydrocolloid molecules that leads to an increase in resistance to flow of sample, and therefore, its viscosity increased (Farahmandfar et al., 2017). Razak et al. (2018) stated that the type and concentration of hydrocolloids significantly affected the viscosity of the mango filling (Razak et al., 2018). The increased viscosity resulted from the gum addition was observed in different food products like frozen milk dessert and ice cream (Chaves et al., 2018). According to the results, five optimized formulations which had higher viscosity were chosen and prepared by varying shahi and zedo gum content based on Table 4.

### 3.2 Quality assessment of optimized samples

#### 3.2.1 CIE L*, a*, b* color values

**FIGURE 2:**
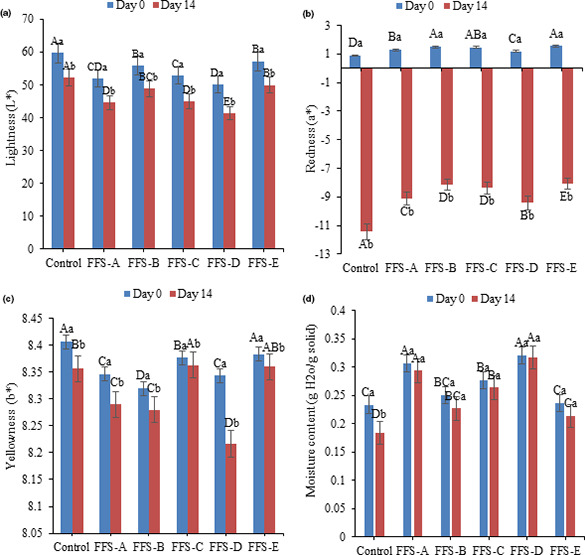
a. Change in the lightness of FFS samples during storage, b. Change in redness of FFS samples during storage c. Change in yellowness of FFS samples during storage d. Change in the moisture content of FFS samples during storage. Different uppercase letters indicate a statistical difference in studied variables among samples at *p* < .05. Different lowercase letters indicate a statistical difference in studied variables among day 0 and day 14 at *p* < .05

Color measurement was done to considerable influence of the color of the products on consumer acceptance. It is the main concern in formulated food with iron because color changes frequently affect the organoleptic properties. The average L*, a*, and b* color data are summarized in Figure 2a–c. The addition of gum to the FFS samples caused the L* and b* values to decrease, it is because of the cloudy properties of gums, particularly in the case of FFS‐D. During storage time, all color indexes were decreased. The L* value of all samples was ranged from 41.33 to 59.71, which proved samples are fairly dark due to presence of iron and also meat powder in the formulation. The positive a* values at first day of storage represent the degree of redness within the color space, and the negative a* values at the end of storage time, which represent the greenness, decreased in control samples, whereas the samples containing higher amount of gum exhibited a higher a* values (Rostami et al., 2018; Taghdir et al., 2017).

#### 3.2.2 Moisture content

The results of moisture content of FFS samples at day 0 and day 14 are illustrated in Figure 2d. As displayed in Figure 2d, during the storage time the moisture content in all FFS samples decreased and it was statistically significant in the control sample. Hydrocolloids molecules by bounding the water modify the properties of the food ingredients. In other words, hydrocolloids are hydrophilic components and retard the loss of the moisture from products during storage (Razak et al., 2018). There is a positive correlation between gum concentration and the moisture content. The FFS‐A, FFS‐C, and FFS‐D samples which had higher gum content exhibited higher moisture content than others. The result is similar to Razak et al. (2018) where the increase in concentration of different types of added hydrocolloids to mango fillings increased the moisture content to 47.48%. Chaves et al. (2018) also observed that the higher concentration of locust bean gum could promote the increase in the moisture content of milk frozen dessert samples (Chaves et al., 2018). Hydrogen bond formed between the polar groups of protein and water is a key factor in water uptake of protein. Oxygen of carbonyl, carboxyl, and hydroxyl groups plays the most important role in the formation of hydrogen bonds with water. Free amine groups can also participate in hydrogen bonding which increased the moisture content of FFS samples containing gum.

#### 3.2.3 Mineral and vitamin contents

**FIGURE 3:**
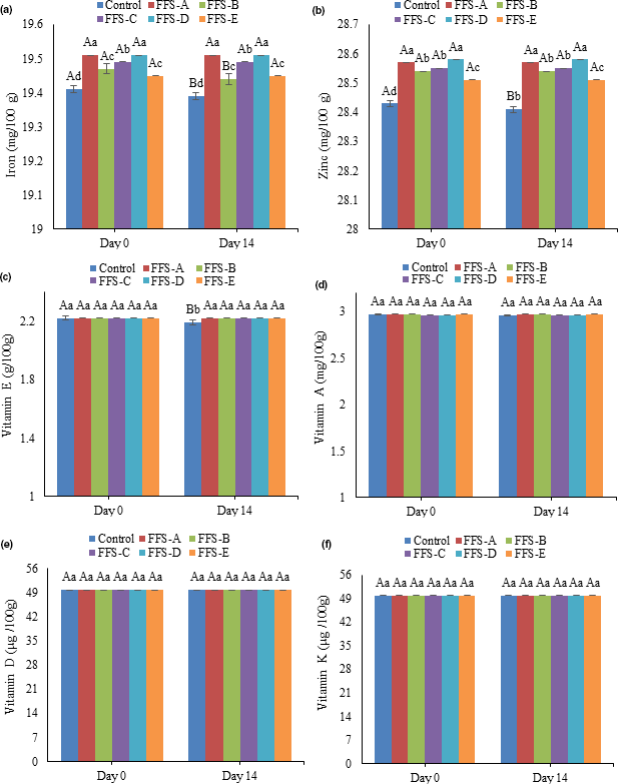
a. Change in iron content of FFS samples during storage, b. Change in zinc content of FFS samples during storage c. Change in vitamin E of FFS samples during storage d. Change in vitamin A of FFS samples during storage. e. Change in vitamin D of FFS samples during storage. f. Change in vitamin K of FFS samples during storage. Different uppercase letters indicate a statistical difference in studied variables among samples at *p* < .05. Different lowercase letters indicate a statistical difference in studied variables among day 0 and day 14 at *p* < .05

The results of change in iron, zinc, and fat‐soluble vitamins are illustrated in Figure 3(a–f). During the storage time, the iron and zinc contents were decreased in control samples, whereas in FFS samples containing hydrocolloids no decrease in mineral was observed. Iron is a mineral and very stable when exposed to light, temperature, heat, extreme pH, and other factors affecting the organic compounds (Sharma et al., 2020). Given that none of these processes have been performed on the samples and they have been stored at 4°C, it can be said that insignificant decrease in iron content of the samples is absolutely logical.

In a study, Fadavi et al. (2014) measured the amount of iron and zinc in zedo gum. The amount of iron in zedo gum samples with different colors was from 7.8–9.3 ppm, and the amount of zinc was 1.2–3.0 ppm (Fadavi et al., 2014). Therefore, the reason for the higher amount of iron and zinc in FFS samples containing zedo gum can be related to the amount of iron in zedo gum. The higher vitamin reduction was observed in control sample, and the highest reduction belonged to vitamin E (*p* < .05). No significant changes were observed in the samples containing hydrocolloids (*p* > .05). Sharma et al. (2020) claimed that more than 94.0% and 92.0% of iron and vitamin A were, respectively, retained in fortified pasta (Sharma et al., 2020).

#### 3.2.4 Sensory properties

**FIGURE 4:**
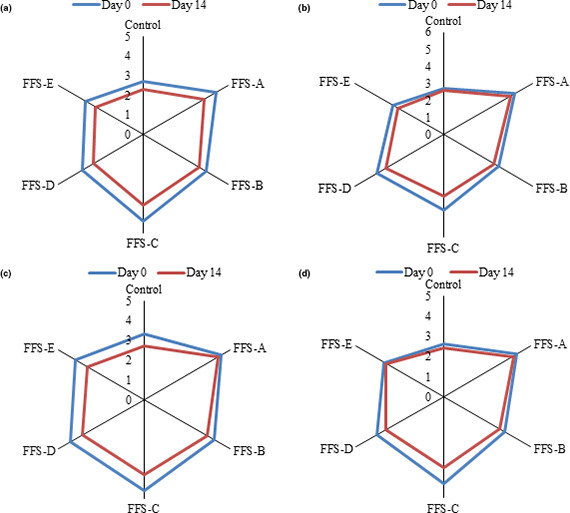
a. Change in the color score of FFS samples during storage, b. Change in taste score of FFS samples during storage, c. Change in appearance score of FFS samples during storage, d. Change in odor score of FFS samples during storage. Different uppercase letters indicate a statistical difference in studied variables among samples at *p* < .05. Different lowercase letters indicate a statistical difference in studied variables among day 0 and day 14 at *p* < .05

One of the challenges in designing a new formulated food is to make it attractive from a sensory point of view. The results of sensory properties of different FFS samples are shown in Figure 4(a–d). In all sensory tests, the mean score of FFS samples decreased over time and the control samples gained the lowest mean scores by panelists. Regarding the color indices evaluated, it was observed that control sample showed the least sensory score. The higher hydrocolloid concentration leaded to the higher mean score of sensorial properties. Although the control samples showed higher lightness than samples containing gums, panelist preferred FFS which contains zedo and shahi gum higher than 0.95 g. The addition of hydrocolloids to the formulation resulted in improving the taste and appearance of FFS samples besides the moisture content. It is because of prevention of emulsion phase separation which caused better mouth feel and appearance. Also, the addition of gums influenced the viscosity of the samples due to greater retention of water, hydration, and level of intermolecular attractions associated with the taste of FFS samples. The absence of syneresis with the addition of hydrocolloids in the emulsion‐based products was proven in previous studies. It seems to be that the phenolic compounds and hydration properties of gums affect the odor of FFS samples. So that control samples had a lower mean odor score. Presumably, the interaction between proteins and polysaccharides occurs due to hydrophobic, electrostatic, and hydrogenic interactions (Nepovinnykh et al., 2019) traps the odor of samples containing hydrocolloids.

## 4 CONCLUSION

The effect of shahi and zedo gum on the calorific value and viscosity of the FFS samples was evaluated using RSM. Shahi and zedo gum were found to be more significant variables affecting the intrinsic viscosity, color, moisture content, and sensorial properties of FFS samples. Antagonistic effects of zedo and shahi gum on vitamins and mineral were not observed and samples containing higher amount of hydrocolloids exhibited higher moisture content, viscosity, color indexes, and sensory scores. Considering that sensory properties should be given priority along with physicochemical characteristics when a new product is designed, the results of this study recommend the use of 1.26 g of shahi gum and 0.95 g of zedo gum to produce formulated food supplement for soldiers.

## CONFLICT OF INTEREST

The authors declare that they do not have any conflict of interest.

## ETHICAL APPROVAL

This study does not involve any human or animal testing.

## INFORMED CONSENT

Written informed consent was obtained from all study participants.
